# Risk of Colorectal Polyps and Malignancies Among Predominantly Rural Hispanics

**DOI:** 10.1007/s10903-018-0802-x

**Published:** 2018-08-11

**Authors:** Gabriela Orsak, Carlton M. Allen, William Sorensen, Paul McGaha

**Affiliations:** 1 Department of Epidemiology and Biostatistics, School of Rural and Community Health, University of Texas Health Science Center at Tyler, 11937 US HWY 271, Tyler, TX 75708-3154 USA; 2 Center for Rural and Community Health, University of Texas Health Science Center at Tyler, Tyler, TX USA; 3 Department of Health and Kinesiology, University of Texas at Tyler, Tyler, TX USA; 4 Department of Community Health, University of Texas Health Science Center at Tyler, Tyler, TX USA

**Keywords:** Hispanic, Colorectal cancer, Colonoscopy, Polyps

## Abstract

Colorectal cancer is the fourth most frequently diagnosed cancer. However, due to variations in diet, it was hypothesized that risk of adenomatous or hyperplastic polyps or malignancies would be lower among Hispanics. Participants (*n* = 1671) underwent a colonoscopy. Results were grouped into one of four groups: normal, hyperplastic polyps only, adenomatous polyps, and malignancies. As expected, Hispanics had a lower risk of hyperplastic (*p* = .031, OR = 0.47) and adenomatous polyps (*p* = .031, OR = 0.66) than non-Hispanic Whites. Comparison between malignancies was not possible as no Hispanics had a malignancy. Contrary to expectations, risk of hyperplastic and adenomatous polyps and malignancies were no different between non-Hispanic Blacks and Whites. Among rural and mostly rural populations, Hispanics had a lower risk of hyperplastic and adenomatous polyps.

## Introduction

Colorectal cancer (CRC) is the fourth most frequently diagnosed cancer in the United States and second most commonly diagnosed cancer in both Hispanic men and women [1, 2]. In 2013 136,119 people were diagnosed with CRC, with 51,813 succumbing to the disease [1]. In the United States, colonoscopy has become the standard for CRC screening, as it significantly reduces mortality [3, 4]. Polyps found in CRC screenings can be divided into the following types: hyperplastic polyps, polyps with no malignant potential, adenomatous polyps, polyps with malignant potential, and malignancies [4]. Those who have adenomatous polyps are at increased risk for developing cancer compared to those without adenomatous polyps or those with hyperplastic polyps [4]. Detection and removal of these precursor lesions prevent many cancers and reduce mortality [4]. Although colonoscopy screenings save lives, screening for minorities, underserved populations, older adults (> 60), men, and the un/under insured are low [5, 6].

With the exception of some small metropolitan statistical areas, East Texas consists of primarily rural communities. It has the highest rates of CRC incidence (52.5–52.7 age-adjusted rate per 100,000 vs. 47.2 for Texas overall) in Texas and one of the highest rates of CRC mortality (19.5–20.6 age-adjusted rate per 100,000 vs. 17.4 for Texas overall) [7]. Non-adherence to cancer screening recommendations, diagnosis of cancer at a later stage, and higher cancer mortality is more likely among rural residents [8–10]. Living in a rural or mostly rural community offers unique challenges. Residents often have to travel long distances to seek care and the number of specialists in the area is often limited. This problem intensifies for older adults or for those who are uninsured. Public transportation is mostly lacking in the area, limiting ones’ ability to return home following the procedure, as driving is restricted for safety reasons. Therefore, seeking preventive services such as colonoscopies may not be a priority for individuals in these communities.

Common risk factors for CRC include smoking, alcohol consumption, and diets low in fiber (e.g. beans) [11]. Hispanic adults have some of the lowest rates of smoking (12.1%) when compared to non-Hispanic Whites (19.4%) and non-Hispanic Blacks (18.4%) [12]. Hispanics also report the lowest rates of alcohol consumption (41.6%) when compared to non-Hispanic Whites (56.7%) and non-Hispanic Blacks (42.8%) [13]. In addition, their diet is richer in fiber than mainstream U.S. diets [14].

Prior CRC-related studies have focused primarily on non-Hispanic White populations, though some studies report lower prevalence of adenomatous polyps in Hispanics [15, 16]. The literature suggests that non-Hispanic Blacks report similar or higher risk of adenomatous polyps when compared to non-Hispanic Whites [16–18], which may be due to lifestyle choices such as smoking. Research is limited in assessing the risk of hyperplastic polyps across cultures, with few having directly compared risk of adenomatous or hyperplastic polyps in Hispanics, non-Hispanic Whites and non-Hispanic Blacks. Literature examining these risk among mostly rural populations is even scarcer. Therefore, this paper sought to examine the risk of hyperplastic and adenomatous polyps, and malignancies among rural or mostly rural Hispanics, non-Hispanic Whites, and non-Hispanic Blacks. Specifically, it was hypothesized that colonoscopy would be less likely to identify hyperplastic (Hypothesis 1), adenomatous polyps (Hypothesis 2), and malignancies (Hypothesis 3) among Hispanics when compared to non-Hispanic Whites. Further, non-Hispanic Blacks would more likely to have a hyperplastic (Hypothesis 4) and adenomatous polyps (Hypothesis 5) and malignancies (Hypothesis 6) identified compared to non-Hispanic Whites.

## Methods

### Participants

**Fig. 1:**
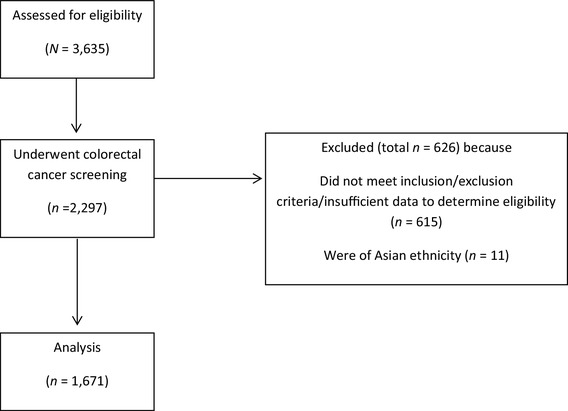
CONSORT diagram

The final sample consisted of 1671 study participants who underwent a colonoscopy (Fig. 1). Descriptive statistics of demographic and medical variables are available in Table 1. The sample was comprised of mostly women (59.6%), persons with insurance (70.5%), and individuals with no family history of colon cancer (79.5%). The mean age was 60.4 years (SD = 6.9) and ranged from age 45–76. The sample consisted of primarily non-Hispanic White participants (64.2%), followed by non-Hispanic Black (25.1%), and Hispanic (10.7%) of participants. This was in contrast to the region that has a slightly larger proportion of non-Hispanic White (67%), and Hispanic (15%) residents, but smaller proportion of non-Hispanic Blacks (15%) [19].

**Table 1:** Medical and demographic characteristics of participants (*n* = 1671)

Variable	Overall	Non-Hispanic White	Non-Hispanic Black	Hispanic
	*n* (%)	*n* (%)	*n* (%)	*n* (%)
Age mean (SD)	60.4 (6.9)	61.1 (6.9)	59.6 (7.2)	57.99 (5.7)
Gender				
Male	675 (40.4)	443 (41.3)	168 (39.9)	64 (36.0)
Female	996 (59.6)	629 (58.7)	253 (60.1)	114 (64.0)
Race/ethnicity		1072 (64.2)	421 (25.1)	178 (10.7)
Insurance status				
Un-/under-insured	493 (29.5)	258 (24.1)	94 (22.3)	141 (79.2)
Insured	1178 (70.5)	814 (75.9)	327 (77.7)	37 (20.8)
Familial history of colon cancer				
No	1329 (79.5)	831 (77.5)	339 (80.5)	159 (89.3)
Yes	185 (11.1)	131 (12.2)	46 (10.9)	8 (4.5)
Missing/did not answer	157 (9.4)	110 (10.3)	36 (8.6)	11 (6.2)

### Instrumentation

Demographic and medical information (i.e. age, gender, race/ethnicity, insurance status, family history of colon cancer, and colonoscopy outcomes) was gathered through patient report and the electronic medical record that were collected at time of colonoscopies. Race/ethnicity was divided into three categories: non-Hispanic White, non-Hispanic Black, and Hispanic. Race/ethnicity was self-reported and birthplace information was not collected, nor was use of Spanish surname lists from the U.S. Census Bureau used for any sort of validation of this variable. Insurance status, gender, and family history of colon cancer were coded as dichotomous variables. Colonoscopy outcomes were divided into four categories: normal, hyperplastic polyp only, adenomatous polyp, and malignancy. Screenings were considered abnormal if participants had one or more biopsied lesions, regardless of the pathology. Lesions were categorized as hyperplastic, adenomatous or cancerous (malignant).

### Procedure

The data analyzed during the current study is available from the corresponding author on reasonable request. Inclusion criteria were as follows: (1) individuals 45–76 years of age, (2) undergoing a colonoscopy, and (3) speaking English or Spanish. Exclusion criteria included (1) being less than 45 years of age, or over 76 years of age, (2) not speaking English or Spanish and (3) not undergoing a colonoscopy, and (4) having previously been diagnosed with CRC in the past. Data was collected through hospital records. Initially, 3635 participants were identified. Of this number, 2297 underwent colorectal cancer screening, but 615 had to be excluded because they did not meet all inclusion/exclusion criteria or insufficient data was available to determine eligibility. Since the primary variable of interest was ethnicity, and participant numbers were too low for appropriate comparison to the three other racial/ethnic groups, participants of Asian ethnicity were excluded from the study (*n* = 11). Analysis was conducted on those who underwent a colonoscopy only (*n* = 1671; Fig. 1). Data was collected between 2014 and 2016. With the exception of some small metropolitan statistical areas, the catchment area consisted of rural communities with little or no access to public transportation.

The study was part of a community outreach program for CRC screening in 19 counties of Northeast Texas organized by the Northeast Texas Center for Rural and Community Health (NETCRCH), University of Texas Health Science Center at Tyler (UTHSCT). Study participants were recruited either by referrals from UTHSCT clinics (general population) or through community outreach events organized by NETCRCH which targeted un- or under-insured individuals. If patients were seen at UTHSCT clinics and met all inclusion/exclusion criteria, they were recommended/referred for colonoscopy or a Fecal Immunochemical Test (FIT) regardless of payer source.

The community-based outreach events organized by NETCRCH involved educating individuals about the guidelines for CRC screening and screening options. Follow-up, scheduling of colonoscopies, and medical record numbers were then provided for eligible individuals. Educational sessions given by community health workers and support staff were held at various community venues and health fairs throughout East Texas to stress the need for routine screenings. The program was promoted through partnerships and outreach events held by community organizations such as churches, workplaces, and barber/beauty shops.

If participants were deemed un- or under-insured and unable to pay for services, screenings were provided free-of-charge and an additional $20 gift card for transportation was provided upon completion of a colonoscopy.

As mentioned above, participants were given the option of undergoing colonoscopy or completing a Fecal Immunochemical test. However, only individuals who underwent colonoscopy screening were analyzed for this paper. Participants were asked to complete a short questionnaire, which included demographics, screening status, health insurance coverage, screening method preference, and family history of colon cancer.

Multiple clinical partners were enlisted to optimize recruitment for CRC screening. Partners included an academic medical center, a charity clinic, health districts, health departments, and federally qualified health centers. All colonoscopy procedures were performed by one gastroenterologist. Polyps identified during colonoscopy were removed. Participants with a biopsy demonstrating a precancerous polyp or cancer were scheduled for clinical follow-up and intervention as appropriate, based on National Comprehensive Cancer Network guidelines [20].

### Statistical Analysis

Data was analyzed using Statistical Package for Social Science (SPSS) version 23 [21]. The hypotheses were tested using a multinomial logistic regression. The outcome variable consisted of four categories (normal, hyperplastic polyp, adenomatous polyp, and malignancy). Categorical variables entered were gender, race/ethnicity, insurance status, and family history of colon cancer. A continuous variable of age was also entered into the model. Of these variables, insurance status, family history of colon cancer, and age were entered into the model as covariates, since prior research has found these groups to have increased risk for abnormal results [6]. The frequencies, descriptive statistics, and distribution of data were examined first. Descriptive statistics were used to assess demographic and medical variables. Skewness, kurtosis, and histograms were used to assess normality for the continuous variable (age). Tests for the assumptions of multicollinearity and linearity of the continuous variable were conducted. The tests did not reveal problems with either assumption. − 2 Log Likelihood, and Pearson and deviance statistics were tested for model fit. Effect size was measured by R^2^ Cox and Snell, Nagelkerke, and McFadden statistics. Odds ratios were reported as well.

## Results

### Frequency of Normal and Abnormal Colonoscopy Results

Table 2 displays the frequency results of colonoscopy for the overall population and by race/ethnicity. The majority had normal results (44.9%). However, a high frequency of adenomatous polyps was observed among all participants (44.4%), with frequencies highest among non-Hispanic Whites (47.1%), followed by non-Hispanic Blacks (42%) and Hispanics (33.7%).

**Table 2:** Frequency results of colonoscopy for the overall population and by race/ethnicity

	Outcome	N (%)
Overall (all races/ethnicities)	Normal	751 (44.90)
	Hyperplastic polyp only	159 (9.50)
	Adenomatous polyps	742 (44.50)
	Malignancy	19 (1.10)
Non-Hispanic Black	Normal	205 (48.70)
	Hyperplastic polyp only	34 (8.10)
	Adenomatous polyps	177 (42)
	Malignancy	5 (1.20)
Hispanic	Normal	104 (58.40)
	Hyperplastic polyp only	14 (7.90)
	Adenomatous polyps	60 (33.70)
	Malignancy	0 (0)
Non-Hispanic White	Normal	442 (41.20)
	Hyperplastic polyp only	111 (10.40)
	Adenomatous polyps	505 (47.10)
	Malignancy	14 (1.30)

### Results of Hypotheses

It was hypothesized that colonoscopy would be less likely to identify hyperplastic (Hypothesis 1), adenomatous polyps (Hypothesis 2), and malignancies (Hypothesis 3) among Hispanics when compared to non-Hispanic Whites. Table 3 displays the results of the analysis including overall model fit, likelihood ratio tests, effect sizes and parameter estimates. The overall model fit was good. However, insurance status, family history of colon cancer, and gender did not show significant − 2 Log Likelihood improvement. As expected, Hispanics were 53% less likely to have a hyperplastic polyp and 37% less likely to have an adenomatous polyp identified than non-Hispanic Whites. Contrary to expectations, malignancies were not detected among Hispanics, and therefore, the likelihood of malignancy among Hispanics was not determined.

**Table 3:** Results of multinomial logistic regression of race/ethnicity predicting colonoscopy outcomes

Variables	− 2 Log likelihood	Χ^2^	df	*p*
Overall model fit information				
Intercept only model	**2994.48**			
Final model	**2938.83**	**55.65**	**18**	< .**001**
Likelihood ratio tests				
Intercept	2938.83			
Age	**2949.78**	**10.94**	**3**	.**012**
Insurance status	2945.82	6.99	3	.072
Family history of colon cancer	3071.87	1.23	3	.746
Gender	2944.24	5.41	3	.144
Race/ethnicity	**2951.97**	**13.13**	**6**	.**041**

Effect sizes	
Cox and snell	.04
Nagelkerke	.04
McFadden	.02

Variable	B (SE)	*p*	95% CI for odds ratio		
			Lower	Odds ratio	Upper
Parameter estimates					
Hyperplasia					
Intercept	− 0.89 (0.96)	.351	0.06	0.41	2.50
Age	− 0.01 (0.01)	.435	0.96	0.99	1.02
Insurance status	0.07 (0.22)	.761	0.69	1.07	1.66
Family history of colon cancer	0.26 (0.31)	.381	0.76	1.30	2.50
Gender	− 0.09 (0.20)	.650	0.60	0.92	1.32
Non-Hispanic Black	− 0.37 (0.23)	.087	0.42	0.69	1.02
Hispanic	− **0.75 (0.34)**	.**031**	**0.21**	**0.47**	**0.88**
Adenoma					
Age	**0.02 (0.01)**	.**006**	**1.01**	**1.02**	**1.04**
Insurance status	− **0.32 (0.13)**	.**010**	**0.55**	**0.72**	**0.94**
Family history of colon cancer	0.02 (0.16)	.934	0.73	1.02	1.38
Gender	0.22 (0.13)	.078	0.97	1.24	1.57
Non-Hispanic Black	− 0.21 (0.13)	.111	0.63	0.81	1.05
Hispanic	− **0.41 (0.20)**	.**031**	**0.45**	**0.66**	**0.96**
Malignancy					
Intercept	− 4.99 (4.43)	.070	< 0.01	0.01	0.46
Age	0.03 (0.04)	.311	0.96	1.03	1.11
Insurance status	− 0.45 (4.20)	.490	< 0.01	0.64	2.46
Family history of colon cancer	− 0.38 (3.83)	.524	0.23	0.68	26,166,747.94
Gender	− 0.30 (1.72)	.585	0.15	0.74	2.06
Non-Hispanic Black	− 0.29 (2.75)	.607	0.12	0.75	2.28

Significant values are given in bold

Results of colonoscopy are compared to normal results; Race/ethnicity compared to non-Hispanic White; Dummy code for Hispanics is not included in malignancy because malignancies were only observed among non-Hispanic Blacks and non-Hispanic Whites

Next, it was hypothesized that non-Hispanic Blacks would more likely to have a hyperplastic (Hypothesis 4) and adenomatous polyps (Hypothesis 5) and malignancies (Hypothesis 6) identified compared to non-Hispanic Whites. However, contrary to expectations, risk of hyperplastic, adenomatous polyps and malignancies did not differ when compared to non-Hispanic Whites.

## Discussion

The present study sought to examine the risk of abnormal colonoscopy outcomes among Hispanics, non-Hispanic Whites, and non-Hispanic Blacks living in a primarily rural area. As hypothesized, Hispanics had a lower risk of adenomatous and hyperplastic polyps when compared to non-Hispanic Whites. However, we failed to provide support for our third hypothesis that stated that Hispanics would report lower risk of malignancies, because no Hispanic participant had a malignancy. Although we failed to provide support, the lack of reported cases suggests a very low risk of malignancies among Hispanics living in a primarily rural area. Past literature has reported that adenomatous polyps were less likely among Hispanics living in urban areas, supporting our findings [16, 17]. However, literature regarding risk of hyperplastic polyps among Hispanics is lacking, and therefore, this paper may have important implications for determining the preferred CRC screening modality among Hispanic individuals.

Contrary to expectations, risk among non-Hispanic Blacks did not differ from non-Hispanic Whites. Although past literature has supported that risk of adenomatous polyps were higher among non-Hispanic Blacks, research has also found no differences among the groups as well [16–18]. This result suggests, that among rural populations, risk among non-Hispanic Whites and non-Hispanic Blacks remain the same.

Bean consumption is part of a traditional diet for many Hispanics and regular bean consumption is more likely among Hispanics than any other race/ethnicity in the United States [22–27]. The protective relationship between dietary fiber in preventing CRC has been hypothesized since the early 1970s [28]. High dry bean intake or total dietary fiber intake reduces the risk of adenoma recurrence [23, 29]. Research suggests several mechanisms to explain the protective role that fiber or high bean consumption may have on CRC. Due to the high fiber and high-resistant starch content, a low glycemic response may be elicited by the consumption of beans as compared to other high-carbohydrate containing food that may help to prevent CRC [30, 31]. Anti-proliferation of human colorectal cells caused by modifying molecules in cell arrest or cell apoptosis may occur with the consumption of non-digestible fraction peptides present in beans [22]. Finally, with increased fiber intake, a dilution of fecal carcinogens, reduced transit time, and bacterial fermentation of fiber to short chain fatty acids with anti-carcinogenic properties may occur [32, 33].

### Limitations

The study had several limitations. Rates of smoking are among the highest in East Texas [19]. Therefore, smoking rates may have been comparable between non-Hispanic Blacks and non-Hispanic Whites. However, this information was not collected and therefore smoking status could not be discerned from the results. Similarly, a dietary assessment of participants was not collected. A potential limitation may have been the self-identification of race/ethnicity. Hispanics often identify as White and/or as multiple races [34]. The researchers could not address this limitation, as self-identification is self-reported by the participant. Sample sizes for ethnic/racial groups were not equal. To avoid issues with unequal sample sizes, Bootstrapping was utilized. Therefore, the results were not biased upon group size. As expected, age predicted adenomatous polyps, but not when participants only had hyperplastic polyps. Finally, with increasing age, the risk of adenomatous polyps were expected to increase, but it is uncertain why the same was not observed with participants with only hyperplastic polyps. Although, results show a significant relationship between insurance status and adenomatous polyps, it is important to note that the overall likelihood ratio tests for insurance status were not significant.

## Conclusion

Overall, adenomatous polyps were found among a very large percentage of the population, suggesting that, through the outreach program, many cancers may have been prevented. Prior research suggests that approximately 10% of adenomatous polyps become precancerous [35]. Nonetheless, the results suggest that more interventions targeting increased colonoscopy testing are warranted in rural areas. Rural residents usually lack access to specialists, have longer waiting times for appointments, and travel long distances to seek care from specialists. These barriers may prevent people from seeking care. In addition, smoking rates are much higher in rural areas, while rates of exercise are lower. Exercise lowers CRC risk by 25–30% while smoking increases risk 32–41% [36, 37]. Rural locations, therefore, suffer several compounding risk factors that can contribute to CRC.

Future research should focus on examining individuals who also underwent a fecal immunochemical test (FIT). FIT testing is less accurate than a colonoscopy, but is much less invasive, thus, preferable to many individuals as a screening tool. In addition, smoking and alcohol status should also be assessed among individuals undergoing screening for CRC. A cost effectiveness report would have been beneficial, especially since the program was based in rural communities and distances to clinics and outreach events were substantial. Such reports are pertinent in order to understand the impact of colonoscopy testing in rural areas. Lastly, we used a combination of outreach events that were both community- and clinical-based. We did not investigate the individual effect of each outreach method. In conclusion, potential links examining why these lower risks occur need further study. In addition, a cost effectiveness report analyzing colonoscopy outreach programs in rural areas in warranted.
